# Effective Activation and Expansion of Canine Lymphocytes Using a Novel Nano-Sized Magnetic Beads Approach

**DOI:** 10.3389/fimmu.2021.604066

**Published:** 2021-02-19

**Authors:** Iwona Monika Szopa, Monika Granica, Joanna Katarzyna Bujak, Agata Łabędź, Maciej Błaszczyk, Chrystal Mary Paulos, Kinga Majchrzak-Kuligowska

**Affiliations:** ^1^ Department of Physiological Sciences, Institute of Veterinary Medicine, Warsaw University of Life Sciences, Warsaw, Poland; ^2^ Department of Physics and Biophysics, Institute of Biology, Warsaw University of Life Sciences, Warsaw, Poland; ^3^ Department of Surgery, Department of Microbiology and Immunology, Emory University School of Medicine, Atlanta, GA, United States

**Keywords:** adoptive cell transfer, canine T cells, cellular immunotherapy, domestic dog model, microbeads

## Abstract

Expansion protocols for human T lymphocytes using magnetic beads, which serve as artificial antigen presenting cells (aAPCs), is well-studied. Yet, the efficacy of magnetic beads for propagation and functionality of peripheral blood lymphocytes (PBLs) isolated from companion dogs still remains limited. Domestic dog models are important in immuno-oncology field. Thus, we built the platform for induction of canine PBLs function, proliferation and biological activity using nano-sized magnetic beads (termed as MicroBeads) coated with anti-canine CD3 and CD28 antibodies. Herein we reveal that activation of canine PBLs *via* MicroBeads induces a range of genes involved in immediate-early response to T cell activation in dogs. Furthermore, canine T lymphocytes are effectively activated by MicroBeads, as measured by cluster formation and induction of activation marker CD25 on canine T cells as quickly as 24 h post stimulation. Similar to human T cells, canine PBLs require lower activation signal strength for efficient proliferation and expansion, as revealed by titration studies using a range of MicroBeads in the culture. Additionally, the impact of temperature was assessed in multiple stimulation settings, showing that both 37°C and 38.5°C are optimal for the expansion of canine T cells. In contrast to stimulation using plant mitogen Concanavalin A (ConA), MicroBead-based activation did not increase activation-induced cell death. In turn, MicroBeads supported the propagation of T cells with an effector memory phenotype that secreted substantial IL-2 and IFN-γ. Thus, MicroBeads represent an accessible and affordable tool for conducting immunological studies on domestic dog models. Similarities in inducing intracellular signaling pathways further underscore the importance of this model in comparative medicine. Presented herein MicroBead-based expansion platforms for canine PBLs may benefit adoptive immunotherapy in dogs and facilitate the design of next-generation clinical trials in humans.

## Introduction

Adaptive immune responses rely on robust clonal expansion of antigen-specific T lymphocytes. Naïve T cells need to be activated by antigen presenting cells (APC), in order to proliferate and differentiate into specialized effector cells (1). In domestic dogs (*Canis lupus familiaris*), similarly to humans, activation process involves recognition of antigenic protein displayed by APCs in the context of the major histocompatibility complex molecule (MHC), known as the dog leukocyte antigen (DLA) for dogs. During cell-to-cell contact, the surface T cell receptor (TCR) binds the antigen presented within the immunological synapse (2, 3). The engagement of TCR is often referred to as “signal 1”, which triggers intracellular signaling pathways leading to activation of genes involved in T cells expansion and biological activity. The TCR is associated with a complex of transmembrane and intracellular proteins (CD3 complex) and co-receptors, such as CD4 and CD8 molecules, which enable signal transduction to the nucleus leading to cytokine production or actin cytoskeletal rearrangement. In addition to TCR-dependent antigen recognition, a costimulatory signal (“signal 2”) is necessary for the activation of naïve T cells (4, 5). One of the most important co-stimulatory receptors is CD28, which is constitutively expressed on canine T lymphocytes (6). Other co-stimulatory molecules such as CD137 (41BB) or inducible costimulator (ICOS) have been shown to be up-regulated on the surface of activated T cells (7, 8). Prolonged recognition of antigen alone, without co-stimulatory signal leads to T cells anergy and development of self-tolerance (9).

The platform enabling extensive propagation of T cells isolated from domestic dogs is important for immunological research as well as for the development of novel immunotherapy in veterinary medicine, and adoptive cell transfer (ACT) therapy in particular. ACT therapy has shown enormous efficiency in eradicating human hematological malignancies, melanomas, gastrointestinal and epithelial cancers (10–12). This treatment involves isolation of T lymphocytes either from peripheral blood or tumor tissue; their activation and genetic modifications, followed by the long-term expansion and re-infusion back to the oncological patients (10). The specificity against malignant cells is acquired by transfecting the T cells with tumor-specific engineered TCRs or chimeric antigen receptors (CARs). The feasibility and safety of ACT therapy have been recently proven in dogs with non-Hodgkin lymphoma (NHL) (13), spontaneous B cell lymphoma (14–16) and glioma (17). Thus, it is warranted to exploit this regimen in other canine malignancies (18). Importantly, ACT therapy in dogs is also of great interest for comparative and translational medicine, due to the opportunity to evaluate novel immune-modulators and adequately assess adverse effects of innovative CAR-based immunotherapies (19, 20). Noteworthily, the companion dog model has been greatly appreciated, firstly by American National Cancer Institute (NCI) and initiation of the Comparative Oncology Program with the goal to enhance studies on large animal models, that could facilitate human clinical trials design, especially in immuno-oncology field (21, 22). Resulting in the formation of the Pre-clinical Cancer Immunotherapy Network for Canine Trials (PRECINCT) as a part of the Cancer Moonshot program. Therefore, in our study we developed a new methodology for activation and expansion of canine peripheral blood lymphocytes (cPBLs) using nano-sized magnetic beads in contrast to previously published Dynabeads-based approach (14, 15). Thus, creating a platform for future investigations of co-stimulatory mechanism and differentiation of canine T lymphocytes.

Human T lymphocytes undergo successful *ex vivo* expansion with magnetic beads coated with agonistic antibody that provided activation “signal 1” and “2” in the presence of IL-2, which is a well-known immune cells growth factor (23). Currently several manufacturers offer commercial kits for the multiplication of human T lymphocytes in clinical settings, e.g. CTS Dynabeads CD3/28 from Invitrogen, magnetic beads MACS GMP TransAct CD3/28 from Miltenyi Biotec and Stage Expamer technology from Juno Therapeutics (24).

Nevertheless, data concerning efficacy of magnetic beads in expansion protocols of T lymphocytes isolated from peripheral blood of domestic dogs still remains limited. Moreover, the optimal culture conditions of canine T cells in terms of activation signal strength and temperature have not been tested. Therefore, we investigated the impact of nano-sized magnetic beads (termed as MicroBeads) coated with anti-canine CD3 and CD28 antibodies on canine T cells activation, proliferation, apoptosis, memory phenotype and cytokine production as well as induction of intracellular signaling pathways. In our work, we have used Miltenyi Biotec MicroBeads instead of previously reported in dog studies Dynabeads-based technique (15, 16). We used nano-sized magnetic beads, because apart from the much small diameter of about 50nm, they are also biodegradable and therefore do not require removal before transfer. It was also shown that magnetic field-enhanced stimulation by nano-sized beads increased murine T cell expansion *in vitro* and after adoptive cell transfer to mice (24–26). Various ratios of T cell to MicroBeads were applied in our study to determine optimal activation signal strength for substantial canine T cell growth in comparison to commonly use plant mitogen—Concanavalin A (ConA). Additionally, we assessed the influence of the temperature in multiple stimulation settings. Importantly, our results indicate this novel platform used on domestic dog model promotes both the proliferation and function (e.g., IFN-γ production) by T lymphocytes.

## Materials and Methods

### Isolation and Activation of Canine Peripheral Blood Lymphocytes

Canine peripheral blood lymphocytes (PBLs) were isolated from fresh whole blood, obtained from healthy client-owned dogs (n=6) that serve as volunteer blood donors at Warsaw Specialist Blood Bank on an institutionally approved protocol. Blood was obtained during routine veterinary procedures and informed consent was received from all dog owners. Only healthy dogs vaccinated against infectious diseases (Parvovirus, Distemper virus, Adenovirus-2, Parainfluenza virus and Rabies) and receiving regular anti-helminthic treatment were included in the study.

Whole anticoagulated blood was diluted 1:3 in sterile phosphate buffered saline (PBS) and immediately subjected to mono-nuclear cell separation on Histopaque 1077 (Sigma-Aldrich, Taufkirchen, Germany) density centrifugation at 800 x g for 30 min at room temperature (RT). After washing with PBS supplemented with 2mM EDTA and 0,5% fetal bovine serum (FBS, Thermo Fisher Scientific, Waltham, USA) cells were treated with erythrocyte lysis buffer (Thermo Fisher Scientific, Waltham, USA) for 5min at RT followed by washing with PBS supplemented 3% FBS. Isolated canine peripheral blood mononuclear cells (PBMCs) were enumerated using automated cell counter and 4% Trypan blue (Countess II Automated Cell Counter, Thermo Fisher Scientific, Waltham, USA). Cells were resuspended in RPMI-1640 GlutaMAX™ medium containing 0,1% HEPES, 10% FBS, 1% Sodium pyruvate, 1% Nonessential amino acids, 1% penicillin and streptomycin (all from Gibco, Thermo Fisher Scientific, Waltham, USA). PBMCs were cultured overnight (37°C, 5% CO_2_) for monocyte/macrophage depletion *via* plastic adherence at a density of 2 x 10^6^ cells/ml in 6-well plates (Corning, New York, USA). Non-adherent canine PBLs were collected next day and counted.

Enriched PBLs were seeded at a density of 1 x 10^6^ cells/ml and activated with nano-sized magnetic beads (terms as MicroBeads) from Miltenyi Biotec (Bergisch Gladbach, Germany) or Concanavalin A (ConA, Thermo Fisher Scientific, Waltham, USA) in multi-well plates (Corning, New York, USA) without agitation. Magnetic beads were coated with cross-linking anti-canine CD3 antibody (clone CA17.2A12, Bio-Rad, Hercules, USA) and anti-canine CD28 agonist (clone 1C6, Functional Grade, eBioscience, Thermo Fisher Scientific, Waltham, USA) at the concentration recommended by the manufacturer. Final concentration was 0.5 µg of each antibody per 1 ml of cell medium containing 1 x 10^6^ PBLs, which was indicated as a 1:1 ratio of T cell to MicroBeads. To activate lymphocytes with different signal strength, cells were incubated at either 1:2, 1:1, 1:0.5, 1:0.25 or a 1:0.125 of T cell to MicroBeads ratio, or with 5μg/ml ConA, a natural mitogen. To compare efficiency of activation using two types of beads, cells were activated independently with MicroBeads or Dynabeads M-450 Epoxy (Invitrogen, Thermo Fisher Scientific, Waltham, USA). Dynabeads were coated with anti-canine CD3 and CD28 antibodies, similarly to MicroBeads. For expansion experiments PBLs were split and supplemented with 10 ng/ml recombinant canine IL-2 (R&D systems, Minneapolis, USA) starting from day 3 of culture. To assess the impact of temperature PBLs were incubated in 33°C, 37°C, 38.5°C, 40°C or 41°C in humidified incubator, 5% CO2 (Sanyo Electric., Ltd., Japan) and visualized using BX60 microscope (Olympus Optical Co., Germany). Cells were harvested on the days indicated and used for gene expression analysis (Real-time PCR), flow cytometry analysis and Western blot assay.

### RNA Isolation, cDNA Synthesis, and Real-Time PCR Analysis

Canine PBLs were harvested 3, 6 and 24 h post activation, washed with sterile PBS and centrifuged at 1,200 rpm for 4 min. Cell pellets were resuspended immediately in 100 µl of Lysis solution and total RNA was isolated using RNAqueous™—Micro Total RNA Isolation Kit (Invitrogen, Thermo Fisher Scientific, Waltham, USA) according to the manufacturer’s recommendations. Samples were DNase treated and RNA concentration was determined using NanoDrop 2000 (NanoDrop Technologies, USA). One microgram of total RNA was transcribed with the High-capacity FG RNA-to-cDNA kit (Applied Biosystems, Thermo Fisher Scientific, Waltham, USA). Quantitative PCR was performed using SYBR Green Select Master Mix (Applied Biosystems, Thermo Fisher Scientific, Waltham, USA) according to the manufacturer’s instructions. Cycling conditions were as follow: initial polymerase activation at 95°C for 2 min, followed by 40 amplification cycles of denaturation at 95°C for 15 s, annealing at 58°C for 15 s and elongation/extension 72°C for 60s in the Stratagene Mx3005P Light Cycler (Agilent Technologies, USA) using MxPro QPCR Software.

The primers for *CD69* and *CD25* were designed *de novo* using PRIMER3 software (free online access, http://bioinfo.ut.ee/primer3-0.4.0/) and checked using an Oligo Calculator (free online access, http://biotools.nubic.northwestern.edu/OligoCalc.html) and Primer-Blast (NCBI database, https://www.ncbi.nlm.nih.gov/tools/primer-blast/). Melting curve analysis was performed for 30 s for each 1°C interval from 58 to 95°C and amplicons were also analyzed using 2% agarose gel electrophoresis. The other primers used in our study (*PTGS2*, *FOS, EGR2, RGS1, GADD45B)* were reported previously by Mortlock et al. (27). *RPS19* and *HPRT* genes were used as a non-regulated reference (housekeeping) for normalization of the target gene expression. The relative mRNA expression was calculated using the comparative Ct method (28) as 2^−ΔCt^, (ΔCt = Ct_reference_ − Ct_target_). The sequences of primers used for qPCR are listed in **Table 1**.

**Table 1:** Sequence of primers used in Real-time PCR.

Gene	Starters sequence (F – Forward, R – Reverse)
*CD25*	F:5’-ACTCCAGATTTCCACAAACACACA-3’ R: 5’-GCTCTTCTTGGCTTCTTACCACT-3’
*CD69*	F: 5’-AGGGTGCTACTCTTGCGTT-3’ R:5’-CAGTAAGGTTGAGCCAGTTGC-3’
*FOS*	F: 5’-GGAACAGGAGACAGACCAAC-3’ R:5’-TAGGGAAGCCACGGACATC-3’
*PTGS2*	F: 5’-ATGGGTGTGAAAGGCAAGAA-3’ R:5’-TGATGGGTAAAGTGCTGGGC-3’
*EGR2*	F: 5’-GCCGTAGACAAAATCCCAGT-3’ R:5’-CCAAGGACCTCTTCTCTCCA-3’
*RGS1*	F: 5’-ATTGAGTTCTGGCTGGCTTG-3’ R:5’-CGTAGGGGTTGGTGCTTTA-3’
*GADD45B*	F: 5’-CTGGTCACGAACCCTCACAC-3’ R:5’-TCAACAGGCTCTGATGCTGG-3’
*HPRT1*	F: 5’-TTATAGTCAAGGGCATATCC-3’ R: 5’-AGCTTGCTGGTGAAAAGGAC-3’
*RPS19*	F: 5’-GTTCTCATCGTAGGGAGCAAG-3’ R: 5’-CCTTCCTCAAAAAGTCTGGG-3’

### Flow Cytometry Analysis

PBLs were washed with sterile FACS buffer (PBS supplemented with 2% FBS). Non-specific antibody binding was blocked by pre-treatment of cells with Fc Receptor Binding Inhibitor Polyclonal Antibody, (eBioscience™, Thermo Fisher Scientific, Waltham, USA) for 20 min at 4°C. Then the cells were resuspended in 100 µl of FACS buffer and stained with a viability dye Zombie aqua-v500 (Invitrogen, Thermo Fisher Scientific) and with mouse primary antibodies for 30 min at RT. To analyze expression level of surface molecules and activation markers the following antibodies were used: anti-canine CD4-APC (clone YKIX302.9), anti-canine CD5-PerCP (clone YKIX322.3), anti-canine CD8α-v450 (clone YCATE55.9) and anti-canine CD25-FITC (clone P4A10) (all purchased from eBioscience™, Thermo Fisher Scientific, Waltham, USA), according to the manufacturer’s recommendation. For memory phenotype staining the rat anti-canine CD44-PE (clone YKIX337.8.7) and CD62L (clone FMC46) were used (Bio-Rad, Hercules, USA). In addition, corresponding mouse and rat IgG1 and IgG2 isotype controls (Bio-Rad, Hercules, USA) were used to determine the level of background surface staining. After incubation with antibodies, cells were washed twice with FACS buffer, centrifuged at 1,200 rpm for 4 min and resuspended in 200 ul of FACS buffer for analysis. Flow cytometry acquisition was performed on a BD FACS Aria II flow cytometer (Becton Dickinson, Heidelberg, Germany). A total of 50,000 events per sample were acquired. Data were analyzed with FlowJo software (TreeStar Inc., Ashland, USA). Lymphocytes were gated with respect to their size and granularity and doublets as well as dead cells were excluded from analysis.

### Cell Proliferation Assay

To assess the expansion of lymphocytes CellTrace™ Far Red Cell Proliferation Kit (Invitrogen, Thermo Fisher Scientific, Waltham, USA) was applied. A stock solution was prepared according to the manufacturer**’**s protocol. Cells were washed with PBS and resuspend in PBS with 0.1% bovine serum albumin (BSA, Sigma-Aldrich, Taufkirchen, Germany). Per 1 mL of cell suspension, 1 µL of stock solution was used. Cells were incubated with CellTrace™ Far Red dye for 15 min at 37°C protected from light with gentle agitation, followed by 5 min incubation in complete cell medium in order to remove any residual dye remaining in the solution. After wash with cell medium, cells were activated with 5 µg/mL ConA or with MicroBeads at different ratios as described above. Cells were harvested before activation (0h) and 2, 4, and 6 days post activation. The cell suspension was fixed for 20 min at RT using Fixation buffer (eBioscience™, Thermo Fisher Scientific, Waltham, USA).

### Cell Apoptosis and Metabolic Activity Assay

In order to analyze activation induced cell death and metabolic activity of PBLs, the Metabolic Activity Dead Cell Apoptosis Kit with C12 Resazurin, Annexin V, and SYTOX™ Green (Invitrogen, Thermo Fisher Scientific, Waltham, USA) was used. PBLs were stimulated using 5 µg/mL of ConA or activated with MicroBeads at different T cell to MicroBead ratios. Cells were cultured in complete cell medium for 24 h. Afterwards, cells were washed in 1X annexin-binding buffer and resuspended at a density of 1x 10^6^ cells/mL. All of the reagents were prepared according to manufacturer’s instructions. Five microliter of Annexin V-APC, 1µL of C12-resazurin-PE and 1µL of SYTOX™ Green working solution was added to each 100µL of cell suspension, followed by an incubation at 37°C (5% CO2) for 15 min. Then cells were resuspended in 500 µL of 1X annexin-binding buffer and analyzed within an hour after staining, using BD FACS Aria II flow cytometer (Becton Dickinson, Heidelberg, Germany). Positive control for necrosis was prepared by incubating cells with 4mM hydrogen peroxide for 2 h at 37°C before staining. C12-resazurin was used to detect metabolically active cells. Non-fluorescent substrate was reduced by these cells to orange-fluorescent C12-resorufin. Cells positively stained by Annexin V were indicated as apoptotic, while SYTOX™ Green-positive cells as dead.

### Cytokine Production Analysis

For cytokine production analysis PBLs were culture for 7 days and then re-stimulated for 4 h with 15 ng/ml Phorbol-12-Myristate-13-Acetate (PMA) and 500 ng/ml Ionomycin (both from R&D systems, Minneapolis, MN) in the presence of Monensin (eBioscience, Thermo Fisher Scientific, Waltham, USA). All were added per the manufacturer’s instructions. After surface staining including rat anti-canine CD4-PE Cy7 (clone YKIX302.9) and rat anti-canine CD8α-Alexa Fluor 647 (clone YCATE55.9) (both from Bio-Rad, Hercules, USA), intracellular staining for cytokines was performed using Fixation and Permeabilization buffers (eBioscience, Thermo Fisher Scientific, Waltham, USA) according to the manufacturer’s protocol. PBLs were fixed for 20 min in RT washed and resuspended in permeabilization buffer for 20 min followed by 30 min incubation with primary antibodies. For intracellular staining mouse anti-bovine IFN-γ-FITC antibody (clone CC302), with confirmed canine cross-reactivity was used, as well as polyclonal rabbit anti-canine IL-2 primary antibody followed by sheep anti-rabbit IgG PE conjugated secondary antibody staining (1:1,000 dilution) (all purchased from Bio-Rad, Hercules, USA). Afterwards, cells were washed with FACS buffer and analyzed using BD FACS Aria II flow cytometer (Becton Dickinson, Heidelberg, Germany).

### Western Blot Analysis

Total protein concentration in whole cell lysates obtained using RIPA (Radio-ImmunoPrecipitation Assay Buffer), supplemented with protease and phosphatase inhibitor cocktail (Pierce™, Thermo Fisher Scientific, Waltham, USA), was determined using a BCA Protein Assay Kit according to the manufacturer**’**s instructions (Pierce™, Thermo Fisher Scientific, Waltham, USA). Aliquots of cell extracts containing 20 μg of protein were resolved through SDS-PAGE. The proteins were subsequently transferred to a nitrocellulose membrane (Pierce™, Thermo Fisher Scientific, Waltham, USA). The membranes were hybridized with appropriate primary antibody: rabbit monoclonal phospho-p44/42 MAPK (ERK1/2) (Thr202/Tyr204), rabbit monoclonal p44/42 MAPK (ERK1/2) (137F5), rabbit monoclonal phospho-p70 S6 kinase (Thr389)(108D2), rabbit polyclonal p70 S6 kinase, rabbit monoclonal RSK1/RSK2/RSK3 (D7A2H), rabbit polyclonal phospho-Akt (S473), rabbit polyclonal Akt, rabbit monoclonal PI3 Kinase p110 δ (D1Q7R), rabbit monoclonal Histone H3 (3H1) and mouse monoclonal β-actin used as a loading control (Cell Signaling Technology, Danvers, USA). Detection of the specific proteins was performed directly on the nitrocellulose membranes using the ChemiDoc MP Imaging System (Bio-Rad, Hercules, USA) and secondary antibody: donkey anti-rabbit IRDye 800 or donkey anti-mouse IRDye 680 (LI-COR Biosciences, Lincoln, USA).

### Statistical Analysis

Statistical analysis of data was performed using GraphPad PrismTM 5.0 (GraphPad Software Inc., San Diego, USA). Comparisons between 2 groups were analyzed using a two-tailed Student’s *t*-test with Welch’s correction for parametric distribution or Mann-Whitney signed-rank test for nonparametric distribution. For comparisons between multiple groups, a One-way ANOVA followed by Tukey multiple comparisons *post-hoc* test or Two-way RM ANOVA was performed (as indicated in the figure legends). Symbols indicate a significant difference between the indicated groups, as follows: *p < 0.05; **p < 0.01; ***p < 0.001.

## Results

### Nano-Sized Magnetic Beads Induce Immediate-Early Activation Responses in Dog T Cells

**Figure 1:**
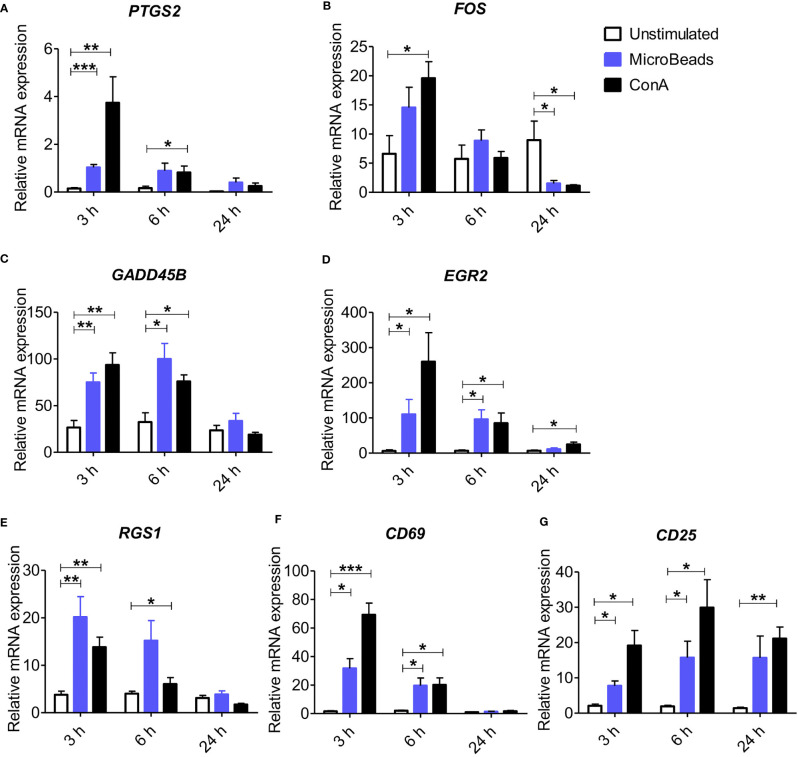
Application of nano-sized magnetic beads coated with anti-canine CD3 and CD28 antibodies (MicroBeads) increases the expression of genes associated with early stages of activation determined by Real-time PCR. Bar graphs showing relative mRNA expression of *PTGS2* **(A)**, *FOS* **(B)**, *GADD45B* **(C)**, *EGR2* **(D)**, *RGS1* **(E)** *CD69* **(F)** and *CD25* **(G)** in unstimulated canine peripheral blood lymphocytes (PBLs) and after stimulation with MicroBeads (1:0.5 T cell:MicroBead ratio) and plant mitogen Concanavalin A (ConA). The analysis was performed using cells from 4 healthy dogs (n=4), in triplicates. Statistical analysis was performed by unpaired Student’s *t*-test (*p < 0.05,**p < 0.01, ***p < 0.001).

To elucidate whether the nano-sized magnetic beads coated with cross-linking antibodies of canine CD3 and CD28 (termed as MicroBeads) could activate canine PBLs, we first determined the early response of genes to T lymphocyte activation. We revealed increased expression of genes previously reported to be involved in immediate-early response in dogs and related to proliferation and cell cycle control (27). These included, prostaglandin-endoperoxide synthase 2 (*PTGS2*), V-FOS FBJ murine osteosarcoma viral oncogene homolog (*FOS*), growth arrest and DNA damage-inducible gene (*GADD45B*), early growth response 2 (*EGR2*) and regulator of G protein signaling (*RGS1*) (**Figures 1A–E**). We also investigated the expression of *CD69* (a membrane‐bound, type II C‐lectin receptor) classical early activation marker of lymphocytes in human and mice (29) and *CD25* (an α chain of receptor for IL-2) that is up-regulated in the late phase of activation (30). To define gene expression kinetics we collected cells 3, 6, and 24 h post activation. All genes increased significantly after 3 h post stimulation with either MicroBeads or ConA (**Figures 1A–E**). Interestingly, while *PTGS2, GADD45B, EGR2* and *RGS1* remained up-regulated after 6 h and returned to the base level of unstimulated cells with 24 h post stimulation, *FOS* decreased significantly in T cells activated with either MicroBeads or ConA upon 24 h of stimulation in comparison to unstimulated cells. We also found that MicroBead-stimulated lymphocytes, similarly to ConA treated cells, exhibit significantly increased *CD69* and *CD25* levels after 3 and 6 h post activation in comparison to unstimulated cells (**Figures 1F, G**). The expression of CD25 remained up-regulated even after 24 h (**Figure 1G**).

### Canine T Lymphocytes are Effectively Activated by Nano-Sized Magnetic Beads

**Figure 2:**
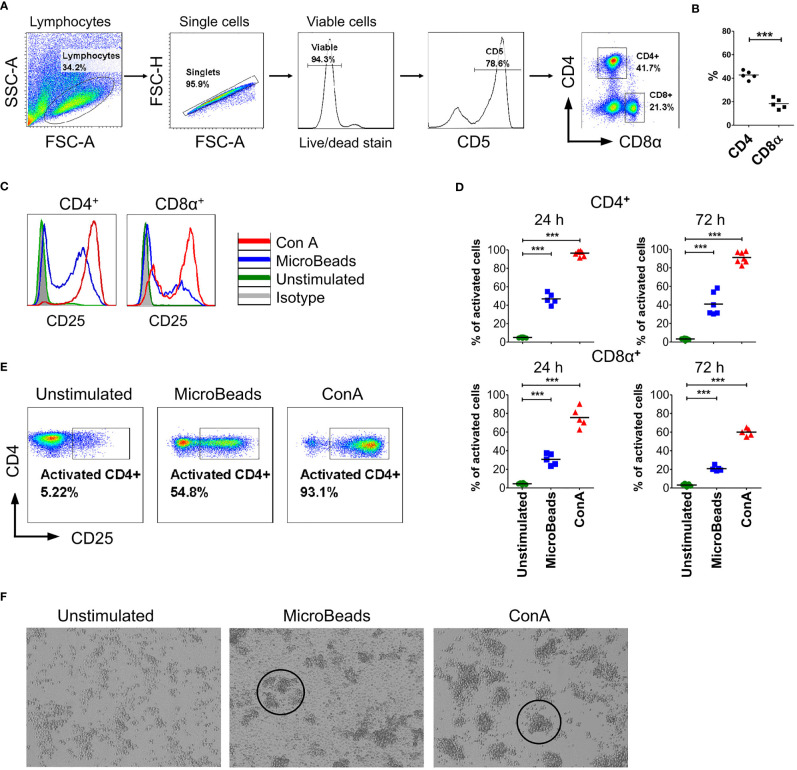
Canine T lymphocytes are effectively activated using MicroBeads. Canine PBLs were analyzed by multicolor flow cytometry (FASC Aria II, Becton Dickinson). **(A)** Gating strategy for flow cytometry analysis. Viable lymphocytes were gated based on FSC and SSC scatter and Zombie dye-v500 staining after doublet-exclusion. Among CD5^+^ T cells two subpopulations were distinguished based on CD4 and CD8α expression. **(B)** Dot graph showing the percentage of CD4^+^ T cells and CD8α^+^ T cells. These two subpopulations were analyzed for activation marker (CD25) expression. Each dot represents one individual dog (n=5). **(C, E)** Representative histograms and cytograms of CD25 expression in unstimulated CD4^+^ and CD8α^+^ T lymphocytes and after stimulation with nano-sized magnetic beads coated with anti-canine CD3 and CD28 antibody (MicroBeads) and plant mitogen Concanavalin A (ConA). **(D)** Quantification of activated CD4^+^CD25^+^ and CD8α^+^CD25^+^ canine T cells 24 and 72 h post-stimulation with MicroBeads (1:1 T cell:MicroBead ratio) and ConA. Each dot represents one individual dog (n=5). Statistical analysis was performed by unpaired Student’s *t*-test (***p < 0.001). **(F)** Representative images from visualization of unstimulated PBLs and after stimulation with MicroBeads or ConA using BX60 microscope (Olympus Optical Co.) (magnify. x100). Canine T lymphocytes were effectively activated by Microbeads and ConA as measured by blast cluster formation (marked in circles) in the initial phase of activation in contrast to unstimulated cells, which exhibit monolayer-like characteristic.

To determine the phenotype and activation status of canine PBLs, we next focused our interest on the surface expression of the activation marker CD25 (α chain of IL-2R). We examined viable T lymphocytes upon 24 and 72 h of stimulation using MicroBeads or ConA. Above 75% of isolated cPBLs were CD5-positive (**Figure 2A**) with the mixture of CD4^+^ (43 ± 3.6%; mean ± SD) and CD8^+^ T lymphocytes (18 ± 4.4%) (**Figure 2B**). Surface staining of CD25 revealed that ConA activate majority of CD4^+^ T cells (96 ± 2.7%), but fewer CD8^+^ T cells (75 ± 10.5%). While MicroBeads activate almost half of CD4^+^ T cells (46 ± 6.1%) and approximately 30% of CD8^+^ T cells 24 h post stimulation (**Figures 2C–E**). With time (72 h post stimulation), CD25 slightly decreased on CD8^+^ T lymphocytes but remained elevated on CD4^+^ T cells (**Figure 2D**). Both MicroBeads and ConA stimulation induced significantly higher expression of activation marker (p<0.001) in comparison to unstimulated cells.

In addition, we evaluated activation status based on phenotypic cells features. As expected, ConA as well as MicroBeads promotes blast cluster formation in the initial phase of activation. Microbeads caused T lymphocytes to form characteristic multiple aggregates, which is a hallmark of T cells activation, while unstimulated cells exhibit monolayer-like characteristics (**Figure 2F**). T cells stimulated *via* MicroBeads were bigger in size during blast transformation. In fact, the average size increased from 7.4 ± 0.22µm (in unstimulated cells) to 10.4 ± 0.8µm in MicroBead-activated lymphocytes, as determined using an automated cell counter and FSC-based flow cytometry analysis (**Supplementary Figure 1**).

By measuring CD25 induction on canine T cells, we compared their activation status when stimulated with nano-sized magnetic beads (MicroBeads) versus stimulated with previously published paramagnetic Dynabeads (14, 15). Paramagnetic Dynabeads contain surface epoxy groups, are larger in size (~270µm) and covalently bind primary amino and sulfhydryl groups in proteins and peptides, thus rendering them useful for coupling antibodies. We found that nano-sized MicroBeads induced CD25 expression on canine T cells to the much higher extend than Dynabeads (**Supplementary Figure 2**). In our study we exploited nano-sized magnetic MicroBeads approach in more detail.

### Activation Signal Strength Affects Canine T Cells Proliferation and Expansion

**Figure 3:**
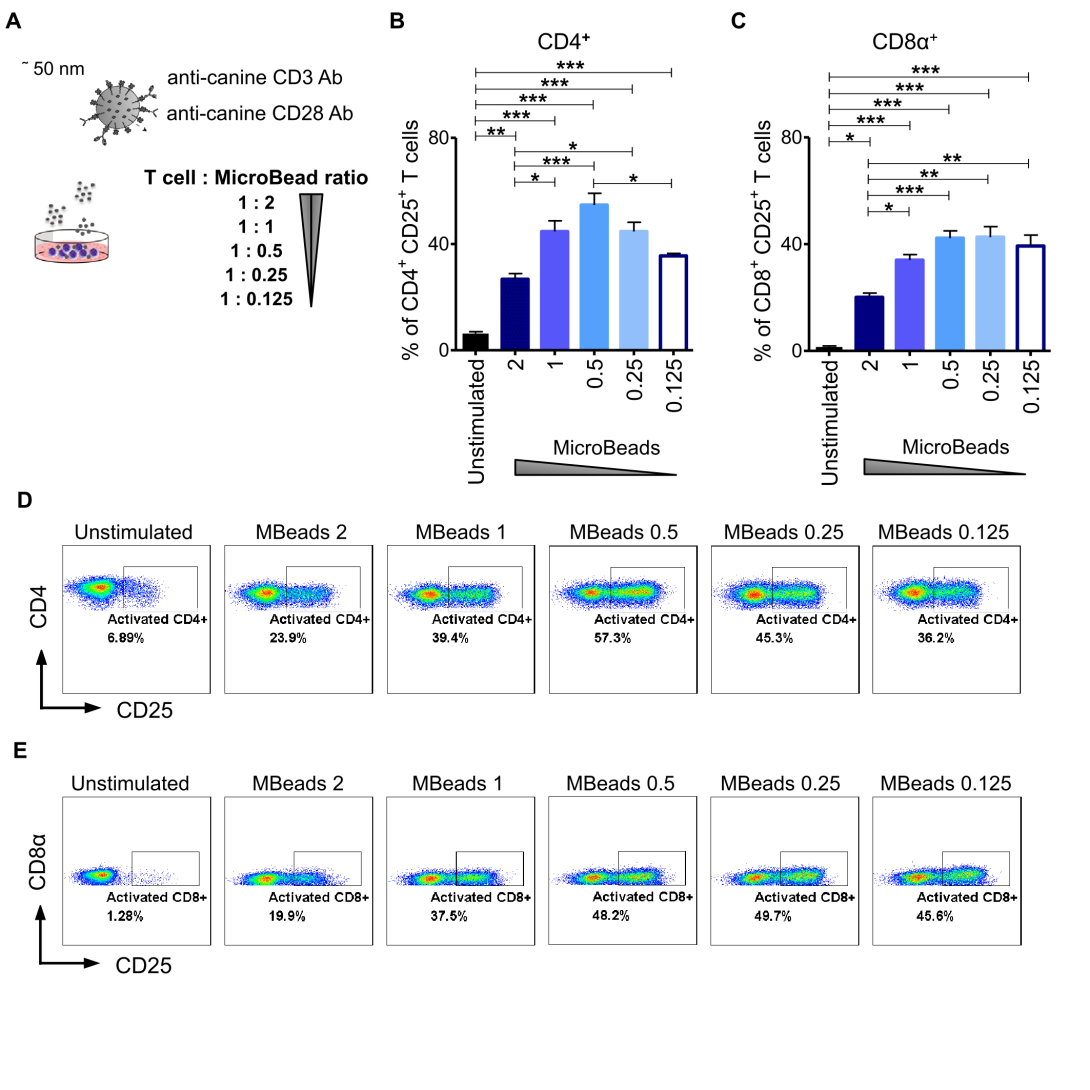
Activation signal strength influence on the expression of CD25 in canine T lymphocyte. **(A)** The graphical scheme of experiment with application of different T cell to MicroBead ratios. Quantification of activated CD4^+^CD25^+^ **(B)** and CD8α^+^CD25^+^ **(C)** canine T cells depending on activation signal strength provided by different MicroBeads concentrations. Data are shown as the mean of five dogs (n=5), and error bars indicate SEM. Statistical analysis was performed by unpaired Student’s *t*-test (*p < 0.05, **p < 0.01, ***p < 0.001). **(D, E)** Representative cytograms of CD25 expression in CD4^+^ and CD8α^+^ T lymphocytes after stimulation with different T cell to MicroBead ratios (FASC Aria II, Becton Dickinson).

To evaluate the impact of activation signal strength on canine T lymphocytes expansion, we stimulated cells at either 1:2, 1:1, 1:0.5, 1:0.25 or a 1:0.125 of T cell to MicroBeads ratio (**Figure 3A**). We hypothesized that using more beads to T cells in the culture would enhance their activation and expansion. To test this idea, we assayed T cells from their expression level of CD25 using flow cytometry. Interestingly, and in contrast to our hypothesis, we discovered that relatively low activation signal strength at the 1:0.5 and 1:0.25 ratio of T cell to MicroBeads was the most effective at inducing IL-2Rα (CD25) expression in CD4^+^ T cells (54 ± 4.4% and 44 ± 6.1% respectively) (**Figures 3B, D**). Furthermore, few MicroBeads concentration (1:0.5, 1:0.25 and 1:0.125 T cell:MicroBead ratio) were needed to activate CD8^+^ T cells with similar efficiency reaching approximately 40% (**Figures 3C, D**).

**Figure 4:**
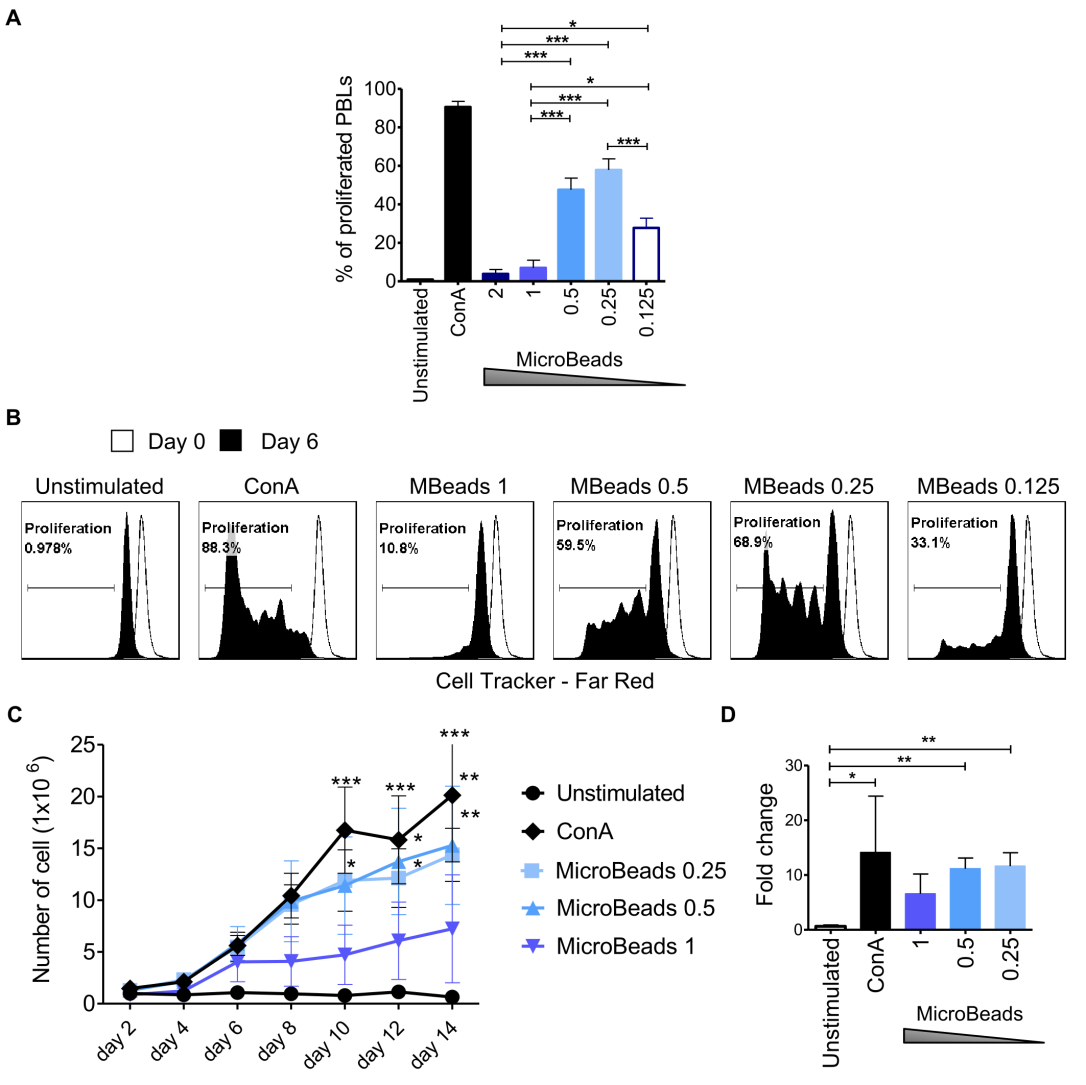
Activation signal strength affects proliferation and expansion rate of canine lymphocyte. **(A)** Quantification of the percentage of canine PBLs proliferation under different activation conditions (T cell to MicroBead ratios as indicated) on day 6 of culture. Data are shown as the mean of three dogs (n=3), and error bars indicate SEM. Statistical analysis was performed by One-way analysis of variance (ANOVA) with Tukey’s Multiple Comparison Test. **(B)** Representative histograms of PBLs stained with CellTrace™ Far Red, cultured with different Microbeads concentrations on day 6 of culture (FASC Aria II, Becton Dickinson). Histograms indicating the multiple peaks at successive generations of cPBLs divisions are shown. **(C)** Expansion kinetic for canine PBLs cultured using different activation signal strength provided by Microbeads and ConA. Data are shown as the mean of four dogs (n=4), and error bars indicate SEM. The number of viable cells was determining every other day for 14 days total using Countess II Automated Cell Counter and 4% Trypan blue. Statistical analysis was performed by Two-way RM ANOVA test (*p < 0.05, **p < 0.01, ***p < 0.001). **(D)** Bar graph showing fold change of cPBLs number upon activation with ConA and different T cell to MicroBead ratios (as indicated) on day 14 of the culture. Data are shown as the mean of four dogs (n=4), and error bars indicate SEM. Statistical analysis was performed by unpaired Student’s *t*-test (*p < 0.05, **p < 0.01, ***p < 0.001).

Next, we used CellTrace™ to determine T cell proliferation post MicroBeads activation at 2, 4, and 6 days post stimulation. Our study revealed that substantial proliferation rate of PBLs was achieved when 1:0.5 or 1:0.25 T cell:MicroBead ratio was used (**Figures 4A–C**). The percent of proliferating T cell were 47.6 ± 6.1% and 57.9 ± 5.8% at 1:0.5 and 1:0.25 T cell:MicroBead ratio respectively, on day 6 of the culture. Interestingly, at higher beads ratios, either a 1:2, 1:1 of T cell to MicroBeads, corresponding to stronger activation signal, we showed that canine T lymphocytes activation and proliferation was attenuated (**Figures 3**, **4**). In addition, to assess cell expansion we counted cells every other day up to 14 days in total (**Figure 4D**). Cell expansion kinetics showed that canine T lymphocytes expanded similarly with ConA and MicroBeads (1:0.5 and 1:0.25 ratios). Moreover, after two weeks, only at 1:0.5 and 1:0.25 T cell:MicroBead ratios, did we find that canine T lymphocytes showed highly significant increase in cell number fold change (p<0.001) (**Figure 4D**).

### Low or Long-Term High Temperature Impairs Canine T Lymphocytes Activation

**Figure 5:**
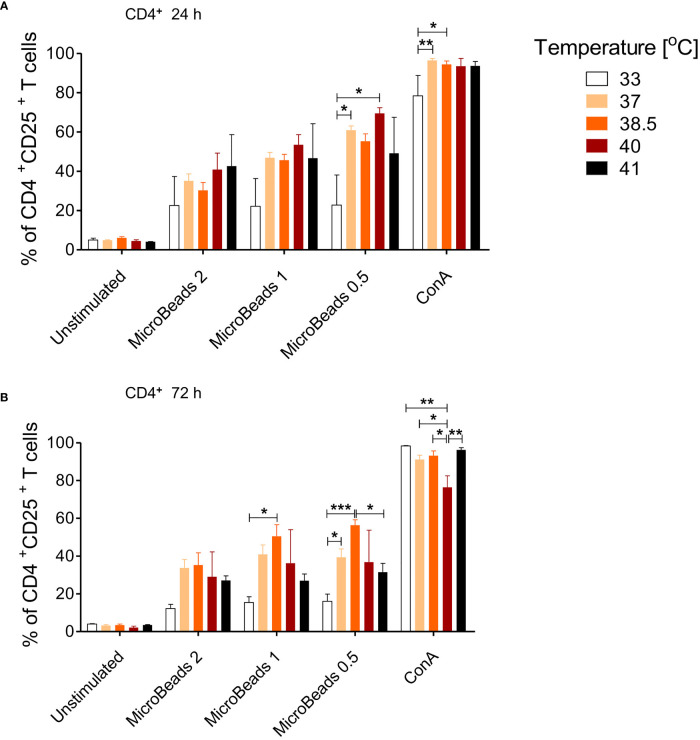
The effect of culture temperature on activation of canine CD4^+^ T lymphocytes. Quantification of activated CD4^+^CD25^+^ T lymphocytes after 24 h **(A)** and 72 h **(B)** post stimulation using different MicroBeads concentrations (as indicated) and ConA. To assess the impact of temperature PBLs were incubated in 33°C, 37°C, 38.5°C, 40°C or 41°C in humidified incubator, 5% CO2 (Sanyo Electric). Data are shown as the mean of five dogs (n=5), and error bars indicate SEM. Statistical analysis was performed by One-way analysis of variance (ANOVA) with Tukey’s Multiple Comparison Test (*p < 0.05 ,**p < 0.01, ***p < 0.001).

Given that canine physiological body temperature varies between 38.3°C and 39.2°C, depending on the dog breed, we sought to next determine the optimal culturing condition of canine T cells in temperatures ranging from 33–41°C. We evaluated activation status in cells stimulated with MicroBeads and ConA for 24 and 72 h. As shown on **Figure 5**, only low temperature (33°C) impaired the activation of canine CD4^+^ T cells 24 and 72 h upon stimulation with either ConA or MicroBeads (**Figure 5A**). Likewise, when T cells were incubated at higher temperatures (40°C and 41°C) for 72 h, their activation was compromised based on reduced CD25 expression (**Figure 5B**). These results however, varied depending on the donor. Similar outcome was observed for CD8^+^ T cells (not shown). Overall, our studies revealed that the optimal temperatures for canine T lymphocytes culture was ~37–38.5°C, based on high CD25 expression. The presented data also revealed that lower signal strength (1:0.5 T cell:MicroBead ratio) induced greater activation of T cells, regardless of the culture temperature *in vitro* (**Figure 5**). For experiments henceforth, cPBLs were cultured at 38.5°C using low-strength activation signal beads (1:0.5 T cell:MicroBead ratio).

### Nano-Sized Magnetic Beads-Based Stimulation Augments the Survival, Function, and Effector Memory Profile of Canine T Cells

**Figure 6:**
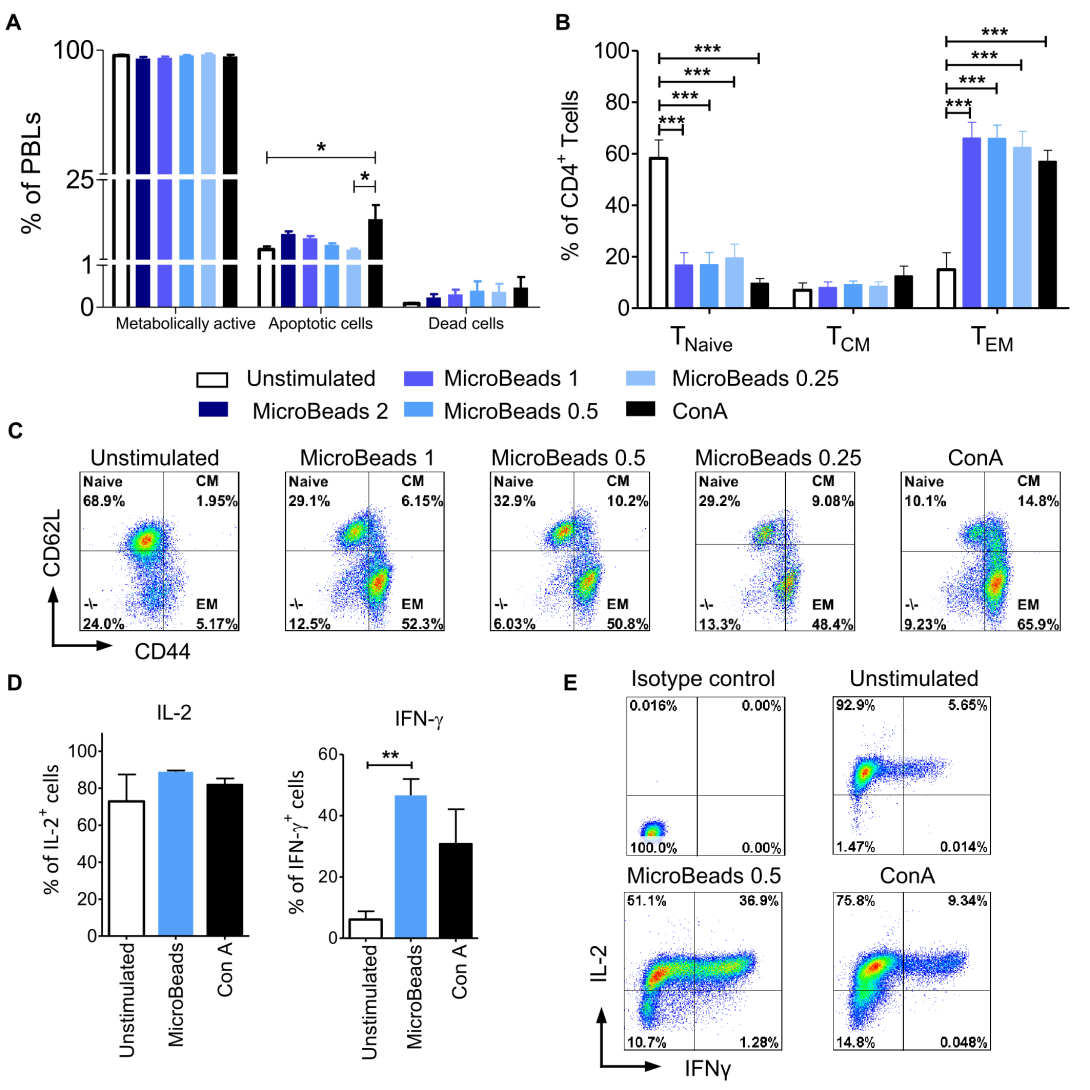
MicroBeads-based stimulation does not increase activation induced cell death (AICD), induces T_EM_ phenotype and IFN-γ expression in canine T lymphocytes. **(A)** Bar graphs showing percentage of metabolically active cells (C12-resorufin^+^), apoptotic cells (Annexin V^+^) and dead cells (SYTOX™ Green^+^) upon stimulation with MicroBeads or ConA stained using the Metabolic Activity Dead Cell Apoptosis Kit. Data are shown as the mean of four dogs (n=4), and error bars indicate SEM. **(B)** Bar graph showing percentage of 1) naïve T cells (T_Naive_, CD44l^ow^CD62L^high^); 2) central-memory T cells (T_CM_, CD44^high^CD62L^high^) and 3) effector-memory T cells (T_EM_, CD44^high^CD62L^low^) upon activation with MicroBeads or ConA after 20 days of culture. Data are shown as the mean of six dogs (n=6), and error bars indicate SEM. **(C)** Representative flow cytometry cytograms of different memory T CD4^+^ cells population stimulated with MicroBeads or ConA (FASC Aria II, Becton Dickinson). **(D, E)** Quantification and representative cytograms of IFN-γ and IL-2 expression in cPBLs activated with MicroBeads or ConA. Data are shown as the mean of three dogs (n=3), and error bars indicate SEM. Statistical analysis was performed by One-way analysis of variance (ANOVA) with Tukey’s Multiple Comparison Test **(A, B)**, and unpaired Student’s *t*-test **(D)** (*p < 0.05, **p < 0.01, ***p < 0.001).

We hypothesized that our optimized culture conditions would improve the survival of canine lymphocytes. Thus, we measured how different concentrations of MicroBeads impacted the viability of canine lymphocytes by measuring apoptosis after their activation. Importantly, we found that, in contrast to ConA-induced activation, canine lymphocytes were healthier when expanded with our MicroBead-based stimulation approach, especially when lower T cell:MicroBead ratio was applied. Our apoptotic assay revealed similar level of apoptosis in 1:0.25 T cell:MicroBead ratio (3.96 ± 0.61%) in comparison to unstimulated cells (3.95 ± 1.92%) (**Figure 6A**). Statistical analysis did not show significant differences in the percentage of apoptotic cells upon activation with different concentration of MicroBeads. T cells stimulated with ConA had higher percentage of apoptotic (13.14 ± 8.53%) in comparison to unstimulated cells. There was no significance difference in percentage of dead cells between different types of activation. Moreover, the percentage of dead cells was low and did not exceed 1.21%. We also determined metabolic activity of PBLs based on reduction of non-fluorescent C12-resazurin into C12-resorufin. All PBLs presented high metabolic activity, without any significant differences between stimulation methods (**Figure 6A**).

Human and murine T cells acquire effector memory phenotype upon stimulation and expansion *ex vivo* (31). To determine memory profile of canine lymphocytes we exploited CD44/CD62L expression pattern, that was previously showed by Rothe et al. (32) to enable division of T cells into three populations: 1) naïve T cells (T_Naive_, CD44^low^CD62L^high^); 2) central-memory T cells (T_CM_, CD44^high^CD62L^high^) and 3) effector-memory T cells (T_EM_, CD44^high^CD62L^low^). Analysis after 20 days of culture revealed that MicroBeads generated T cells with an effector memory (T_EM_) phenotype (up to 65% of cells) compared to unstimulated cells (**Figures 6B, C**).

Intracellular staining was used to assess the amount of effector cytokines: interferon-γ (IFN-γ) and IL-2, produced by cPBLs. Cytometric analysis showed that only MicroBead-activated T cells produced significantly more IFN-γ (p <0.001) in comparison to unstimulated cells; 46.6± 5.4% in comparison to 6.1 ± 2.7%, respectively (**Figures 6D, E**). Interestingly, after activation with ConA and MicroBeads T cells produced comparable high amount of IL-2 compared to unstimulated cells; 81.9± 5.8%, 88.8 ± 1.2% and 72.8 ± 14.6%, respectively (**Figures 6D, E**).

### Nano-Sized Magnetic Beads Induce PI3K Signaling and Phosphorylation of p70 S6 Kinase

**Figure 7:**
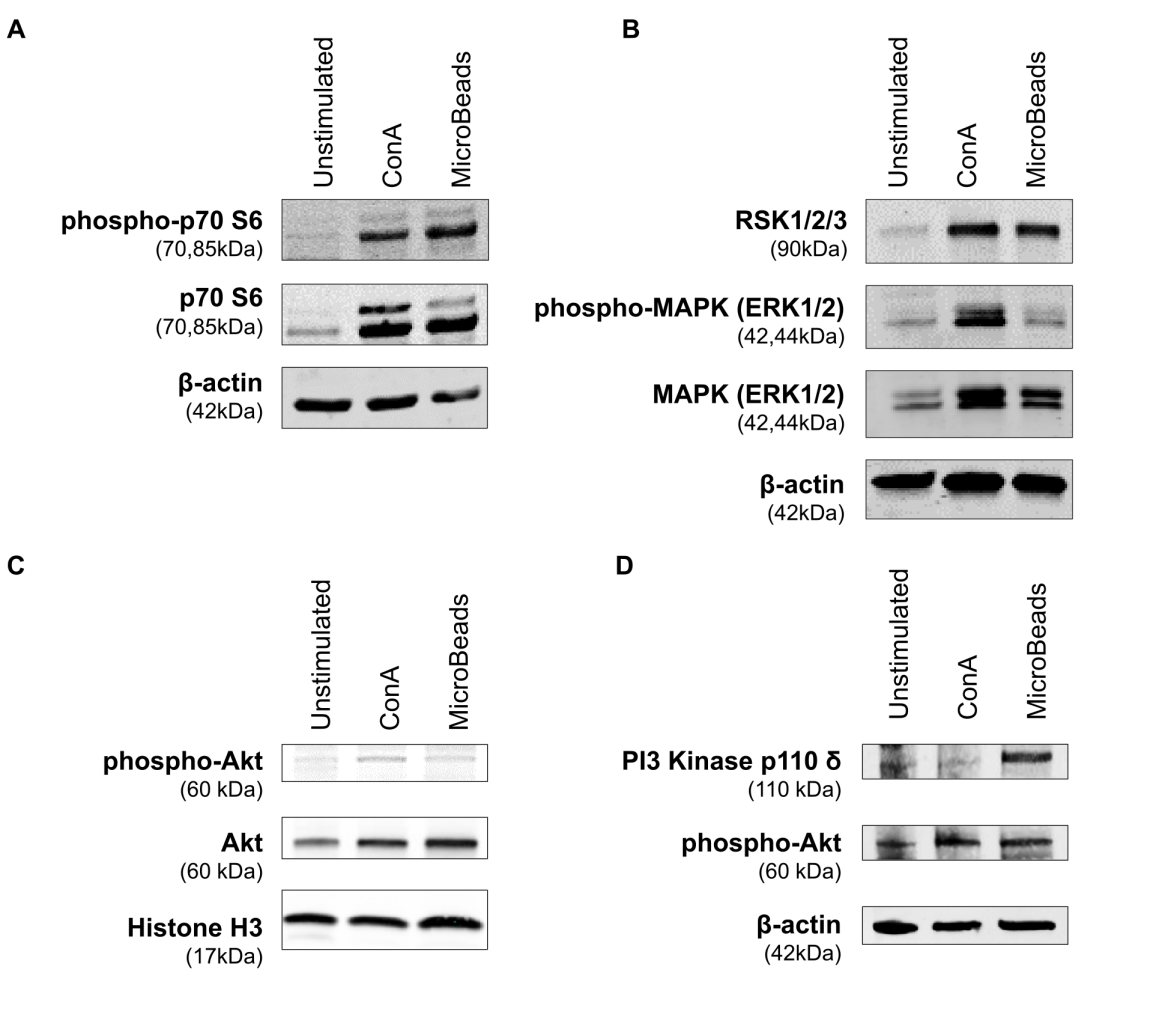
Application of MicroBeads induce PI3K pathway. Western blot analysis of **(A)** phospho-p70 S6 Kinase, p70 S6 Kinase and β-actin (loading control) expression after 1h post stimulation with ConA or MicroBeads, **(B)** RSK1/2/3, phospho-MAPK (ERK1/2), MAPK (ERK1/2) and β-actin (loading control) expression after 1 h post stimulation with ConA or MicroBeads, **(C)** phospho-Akt, Akt and Histone-3 (loading control) expression after 1 h post stimulation with ConA or MicroBeads, **(D)** phospho-Akt, PI3 Kinase p110 δ and β-actin (loading control) expression after 15 min post stimulation with ConA or MicroBeads. Results from one experiment with no replicates are shown.

In order to evaluate the intracellular pathway induction by MicroBeads, we assessed the expression of proteins downstream of the mTORC1 pathway, including p70 S6 kinase [also known as ribosomal protein S6 kinase beta-1 (S6K1)]. MicroBeads as well as ConA induced the phosphorylation of p70 S6 kinase in cPBLs 1 h post activation (**Figure 7A**), which may lead to protein synthesis at ribosomes. We also investigated the expression of proteins involved in mitogen-activated protein kinase (MAPK) pathways (such as ERK1/2 and RSK1/2/3). Phosphorylation of p44/42 MAPK (ERK1/2) kinase was increased by ConA treatment, but less by MicroBeads-based stimulation (**Figure 7B**). RSK1/2/3 was also upregulated 1 h post activation using CoA and MicroBeads in comparison to unstimulated cells (**Figure 7B**). Finally, we evaluated the expression Akt kinase 1 h post cPBLs stimulation and we found it was elevated in T cells stimulated with ConA and MicroBeads compared to unstimulated cells (**Figure 7C**). Also, we sought to investigate earlier time points and found significant phospho-Akt (S473) expression 15 min post activation using ConA and MicroBeads (**Figure 7D**). Interestingly, we also found that MicroBeads activation, but not ConA-based stimulation, induced the expression of PI3 kinase p110 δ subunit in canine T cells (**Figure 7D**).

## Discussion

Similar to humans, companion dogs develop a variety of different allergic and autoimmune diseases as well as different cancers types associated with immune system disorders. Therefore dog models offer multiple advantages in clinical research, ranging from the basic biology of the canine immune system to the evaluation of efficacy and safety of novel cellular immunotherapies in comparison to xenograph mouse model (reviewed in 19, 21, 22, 24, 33, 34). However, stimulation and expansion protocols of T lymphocytes in dogs are not as extensively investigated as in humans or mice systems. Herein, we provide comprehensive and insightful data concerning optimization of canine T lymphocytes activation and proliferation *in vitro*, in the context of activation signal strength and temperature, using antibody-coated nano-sized magnetic beads (MicroBeads). We also evaluated the beads-based expansion platform for the future use in basic and translational immunological studies.

Initially, canine T lymphocytes have been stimulated short-term *via* potent mitogens lectins, such as ConA (35, 36) and phytohemagglutinin (PHA) (37, 38) or longer-term using soluble or plate-bound anti-canine CD3 antibody alone (39–41) or in combination with CD28 antibody (6) along with IL-2 supplementation. However, naïve T lymphocytes require “two signals” of activation provided solely by APCs. Therefore, in human immunological research T cells are expanded *ex vivo* using different types of APCs (42). Several expansion protocols for human T lymphocytes harness natural patients-derived dendritic cells (DCs) generated from peripheral blood monocytes. Other common approach is the use of human immortalized erythroleukemic K562 cells, which serve as artificial APCs (aAPCs) (42). The K562 cells express adhesion molecules that ensure immunological synapse formation and transduction of activation signal. Additionally, the K562 cells might be genetically engineered to express multiple co-stimulatory molecules and cytokines (43). Importantly, human T cells are also very efficiently and commonly expanded, in research and in clinical trials, using antibody-coated bead based DCs. This approach exploit paramagnetic or magnetic beads coated with CD3 and CD28 or other co-stimulatory agonistic antibodies (44–46).

In veterinary settings O’Connor and colleagues (13) were the first that took advantage of artificial APCs stimulation to expand canine T lymphocytes derived from peripheral blood of healthy donors and advance stage NHL-bearing dogs. Using γ-irradiated K562 cells expressing array of human co-stimulatory and adhesion molecules, loaded with anti-human CD3 (clone OKT3), they achieved the canine T cells propagation necessary for adoptive immunotherapy. Moreover, hrIL-21 along with hrIL-2 have been used to ensure selective canine CD8^+^ T cells proliferation. Adopting this method Mata et al. (47) established the canine PBMCs expansion procedure using, in addition to γ-irradiated K562 cells, the mitogen PHA (instead of OKT3) as well as hrIL-21, which resulted in approximately 100-fold expansion of lymphocytes within 2 weeks of culture *in vitro*. This method was further improved by Panjwani and co-workers (2016, 2019) (14, 15), who apart from using beads-based activation, employed also K562 cells transduced to express canine CD86 loaded with agonistic anti-canine CD3 antibody in the presence of cytokine regimen. These authors reported, to date, the largest expansion of T cells (more then 200-fold) derived from healthy companion dogs as well as from patients with refractory B cell lymphoma (14). While these methods are exciting and informative to the field, our work is the first to use nanoparticles to expand canine lymphocytes. Future experiments using IL-21 or other homeostatic cytokines shall be interesting to uncover the factors needed to bolster the generation of canine lymphocytes for clinical use.

Despite impressive efficiency of the K562 aAPCs-based expansion, this platform is like not ideal for wide used in veterinary adoptive immunotherapy in the clinic (42). This limitation is mainly due to the fact that K562 cells origin from human malignant tumor cells (43). Although K562 cells are irradiated prior to co-culture with PBMCs and were not detected upon T cells expansion (43), infusing them to canine patients might raise concerns. Even though K562 cells are not present in the transferred cells population, are harmless for the dogs, and they will meet all manufacturing practice requirements for clinical use, those cells still need irradiation. Such procedure might not be readily available in biomedical research facilities (48) and is unlikely to be used in veterinary clinics or laboratories. Hence our finding that nanoparticles can effectively expand and activate canine lymphocytes has far reaching and immediate implications in the clinic.

Indeed beads-based stimulation is far more accessible and affordable option for expansion of canine T lymphocytes for cellular immunotherapy in veterinary medicine. Research group of Mason and co-workers attempted to make use of anti-canine CD3/CD28 antibodies-coated magnetic Dynabeads (14, 15). They evaluated parallel impact of ConA, K562 aAPCs and beads-based stimulation on canine T cells activation and proliferation *in vitro*. While all three stimuli induced lymphocytes division by day six of culture, only K562-based aAPCs stimulation significantly foster the expansion of T cells from all donors. Beads-based stimulation works well in some donors, but fail in others (14). Interestingly, in subsequent studies, lymphocytes activated with Dynabeads coated with CD3/CD28 antibodies were characterized by higher effectiveness of lentiviral transduction. Hence, there is a rationale for using this method in the development of CAR T cell therapy (15). Noteworthy, similar observations were made in human clinical trials, where some patient’s T cells did not respond to signal provided by the beads (49). Nevertheless, the beads-based stimulation was not abandoned in human medicine. In fact, the beads-based system was shown to be feasible and reliable for FDA-approved clinical applications (44, 49). Therefore, activation platform based on magnetic beads need to be fine-tuned and more deeply investigated in veterinary settings. In our research, we discovered that nano-sized anti-canine CD3/CD28 magnetic beads activation supports proliferation and function of T lymphocytes of healthy donors. We were also the first to discover the optimal activation signal strength. We revealed that relatively low signal strength (1:0.5 and 1:0.25 ratios of T cell to MicroBeads) is beneficial for CD4^+^ and CD8^+^ canine T cells proliferation and acquiring of effector phenotype, regardless of culture temperature.

Several studies suggest that optimizing the bead to T cell ratio should be considered while developing expansion protocols, in order to limit T cells anergy and preserve high cells viability as well as to reduce costs of T cells culture *ex vivo* (45, 46). We obtained optimal effects using relatively low signal strength provided by MicroBead-based stimulation. We speculate that the discrepancies between our results and those already reported (14) might be due to the use of lower T cell to beads ratio (1:3 T cells to beads ratio by Panjwani et al., 2016) and/or due to smaller size of beads exploited in our study. We applied the nano-sized beads (~50 nm diameter), which are around 90-times smaller than classical CD3/CD28 activation Dynabeads (4.5 µm diameter) and thus were suspected to be superior at expanding T cells *ex vivo* (42). These MicroBeads could better mimic immunological synapse and are found to be biodegradable. Moreover MicroBeads could be coated with different co-stimulatory molecules providing the most beneficial signal for desired T cell subpopulation e.g. the ICOS-mediated co-stimulation supports Th17 cells differentiation (50).

Moreover, our results concerning T cell to MicroBeads ratio are in agreement with a research conducted on human PBMCs, which revealed that fewer beads could be used to support antigen-specific T cells survival and proliferation (45, 46). Recently, Martkamchan et al. (45) defined and confirmed that the optimal human PBMCs to anti-CD3/CD28 beads ratio is 1:0.5. Under these conditions the expansion of more than 1,000-fold was achieved within 3 weeks of culture while preserving high cell viability (45). Importantly and similarly to our findings, the lower strength of the signal from beads stimulation led to the expansion of CD8^+^ T lymphocytes. Whereas increase in the signal strength supported CD4^+^ T lymphocytes proliferation. Our research confirmed that canine T lymphocytes react to various signal strength similarly to human T cells, a finding that has immediate implications in further immunological studies in the context of cancer and infectious disease.

It was established that human T cells “fitness” is shaped by activation signal strength and duration (51). Prolonged stimulation of an individual human Th1 cell with a high concentration of the anti-CD3 antibody increased level of anergy of these cells (52). Moreover, signal strength among others factors such as co-stimulatory molecules and cytokine cue, affect T cells differentiation (53, 54). Weak TCR signal promotes Th2 and Th17 response, whereas strong TCR signaling leads to Th1 predominance and is required for Tfh differentiation (53, 55). By using different beads concentrations, activation signal strength can be regulated. Therefore, the employment of MicroBead-based stimulation shall pave new ways for more detailed studies on the differentiation of canine T cells into various subpopulations.

To optimize the activation of canine T cells *in vitro*, we cultured cells in different temperatures ranging from 33–41°C. Cytometric analysis of CD25 marker expression showed that a high level of activated T lymphocytes was obtained by growing cells at 38.5°C, which is a mean physiological body temperature in dogs (56). Culturing PBLs at 37°C or 40°C did not result in significant changes in CD25 expression in comparison to 38.5°C, suggesting that canine lymphocytes can be also cultured at these temperatures. Nevertheless, observations made during our study indicate that while culturing freshly isolated cells is possible at 37°C, T cells that had been subjected to freezing did not activate at this temperature (data not shown). At higher temperatures (40°C, 41°C) canine T cells have not expanded (data not shown), probably due to spontaneous apoptosis. We suspect that based on human data demonstrating that heat stress sensitizes T cells to Fas receptor-mediated apoptosis (57). However, it is necessary to perform further research to fully understand this phenomenon in dogs. In our study, canine T lymphocytes were grown with optimal efficacy at temperature ranging from 37–38.5°C. According to literature data, only Mata et al. (47). have cultured canine CAR modified T cells at higher (though physiological for dogs) temperature of 38.5°C for the use in adoptive cellular immunotherapy (47). Other studies on canine T cells cultured *in vitro* were carried out at a standard incubation temperature of 37°C (13, 15, 16, 36, 58), which we also found is optimal for immunological studies in dogs. Importantly our research, as well as others, on murine and humans T cells (59), revealed that low temperature of 33°C significantly impairs canine T cells activation.

Other elements controlling the expansion and differentiation of T lymphocytes after the recognition of a foreign antigen *in vivo* is the induction of apoptosis, a process referred to as activation-induced cell death (AICD) (60, 61). Mobilization of the TCR receptor provides a strong survival signal for T lymphocytes, but at the same time initiates a series of molecular processes leading to apoptosis. For example by induction of Fas surface death receptor (CD95) and its ligand (CD95L) expression, inhibition of the anti-apoptotic proteins activity and trigger a cascade of caspases (60, 61). We revealed that MicroBeads, contrary to ConA, do not increase apoptosis upon activation and preserve their metabolic activity. Presumably due to the fact that the magnetic beads provide CD28 mediated co-stimulation, which inhibit apoptosis in humans, mainly by activating the PI3K/Akt pathway and promoting anti-apoptotic Bcl-2 and Bcl-xL proteins expression (61, 62).

In studies on the human CD4^+^ T lymphocytes expansion, the advantage of magnetic beads activation over stimulation with only soluble anti-CD3 antibody was demonstrated, both in terms of proliferation rate and the production of cytokines such as IFN-γ and TNFα (24). Moreover, magnetic bead-based stimulation rather than soluble antibody-related activation is currently used in clinical trials for the expansion of tumor-specific CAR T cells (63). We were the first to demonstrate that activation of canine PBLs using MicroBeads not only induces effector memory phenotype of expanded T cells but also enhances production of IL-2 to the levels achieved using ConA and causes significantly greater release of IFN-γ. This function data is an important advantage in the propagation of T cells for clinical grade adoptive cell transfer for cancer and infectious disease.

Finally, we determined the intracellular signaling pathway induced by MicroBead-based stimulation. We found that, similarly to human study (64), MicroBeads induced phosphorylation of downstream proteins of PI3K pathway, and p110 δ subunit of PI3 kinase. Mitogens such as ConA lead to activation of the ERK1 and ERK2, which then phosphorylate and activate downstream targets such as the ribosomal S6 kinases (RSKs). RSK family members are exclusively activated by the ERKs. ERK1/2 and RSK1/2 can also translocate into the nucleus where, they cause transcriptional activation of genes, including transcription factors that control the cell cycle, cell survival and/or cell differentiation (65). In contrast to ConA, which as expected leaded to significant MAPK pathway activation, stimulation with MicroBeads caused phosphorylation of ERK1/2 to much lower extent. However, MicroBeads activation induced phosphorylation of p70 S6 kinase (Thr389), which is required for the action of phosphoinositide 3-dependent protein kinase 1 (PDK1). The PDK1 has a crucial function in T cell activation by nucleating the assembly of a signaling complex in response to CD28 engagement (66).

In conclusion, our study reveals a novel methodology for the activation and expansion of canine PBLs. The ultimate goal of this innovative approach is to uncover basic immunological biology of T cells in domestic dogs and in the near future exploit it for adoptive cell transfer therapy research. Our culturing system enables the swift regulation of T cells activation signal strength, co-stimulatory mechanism and proliferation as well as functionality is a valuable tool for further investigations in the veterinary immunology field. Similarities in inducing intracellular signaling pathways further underscore the utility of companion dog model in comparative medicine. Our new MicroBead-based canine lymphocytes activation and expansion platform has the potential to benefit adoptive immunotherapy in dogs and facilitate the design of next-generation clinical trials in humans.

## Data Availability Statement

The raw data supporting the conclusions of this article will be made available by the authors, without undue reservation.

## Author Contributions

IS: elaboration of research concept and design, creation of scientific hypothesis and determination of research objectives, development of methodology, performing experiments (cPBMCs isolation, cell cultures, T cells activation and proliferation assay, cell expansion, RNA isolation and Real-time PCR analysis, flow cytometry analysis, apoptosis assay, cytokine production and memory phenotype analysis), analysis of the obtained data (including statistical analysis), interpretation of the results, writing original draft, review and editing, figure preparation. MG and JB: development of methodology, performing experiments (cPBMCs isolation, cell cultures, T cells activation, Western blotting). AŁ: performing experiments (proliferation assay). The others are correct. MB: performing experiments (Real-time PCR, Western blotting). CP: supervision, writing original draft, review and editing. KM-K: conceptualization, formal analysis, funding acquisition, supervision, figure preparation, writing original draft, review and editing. All authors contributed to the article and approved the submitted version.

## Funding

This work was financed by the grant No. POIR.04.04.00-00-3EE9/17-00 entitled “*Modification of signaling pathways of canine Th17 lymphocytes subset to improve adoptive cellular immunotherapy for humans*” carried out within the First TEAM programme of the Foundation for Polish Science co-financed by the European Union under the European Regional Development Fund, Smart Growth Operational Programme 2014-2020 (to KM-K).

## Conflict of Interest

The authors declare that the research was conducted in the absence of any commercial or financial relationships that could be construed as a potential conflict of interest.

## Supplementary Material

Supplementary Figure 1 Activation with MicroBeads as well as ConA causes an increase in cell size during blast transformation. Representative flow cytometry cytograms **(A)** and histograms **(B)** indicating cell size based on FSC scatter. **(C)** Dot graph showing changes in size of T cells activated with MicroBeads and ConA. Data obtained using Countess II Automated Cell Counter. The average size increased from 7.4 ± 0.22µm in unstimulated cells to 10.4 ± 0.8µm in MicroBeads activated lymphocytes. Data are shown as the mean of six dogs (n=6), and error bars indicate SEM. Statistical analysis was performed by unpaired Student’s *t*-test (****p* < 0.001).

Supplementary Figure 2 Canine T lymphocytes activation efficiency after application of two types of beads (Dynabeads and MicroBeads 1:0.5) coated with anti-canine CD3 and CD28 antibodies. Dot graph showing the percentage of activated CD4^+^CD25^+^ **(A)** and CD8α^+^CD25^+^ **(B)** T cells. Each dot represents one individual dog (n=4). Statistical analysis was performed by One-way analysis of variance (ANOVA) with Tukey’s Multiple Comparison Test (***p* < 0.01, ****p* < 0.001).
