# Supplementary figures and images for: Effective Activation and Expansion of Canine Lymphocytes Using a Novel Nano-Sized Magnetic Beads Approach
Source: Front Immunol. 2021 Feb 19;12:604066. doi: 10.3389/fimmu.2021.604066 (PMC7933476; doi:10.3389/fimmu.2021.604066)

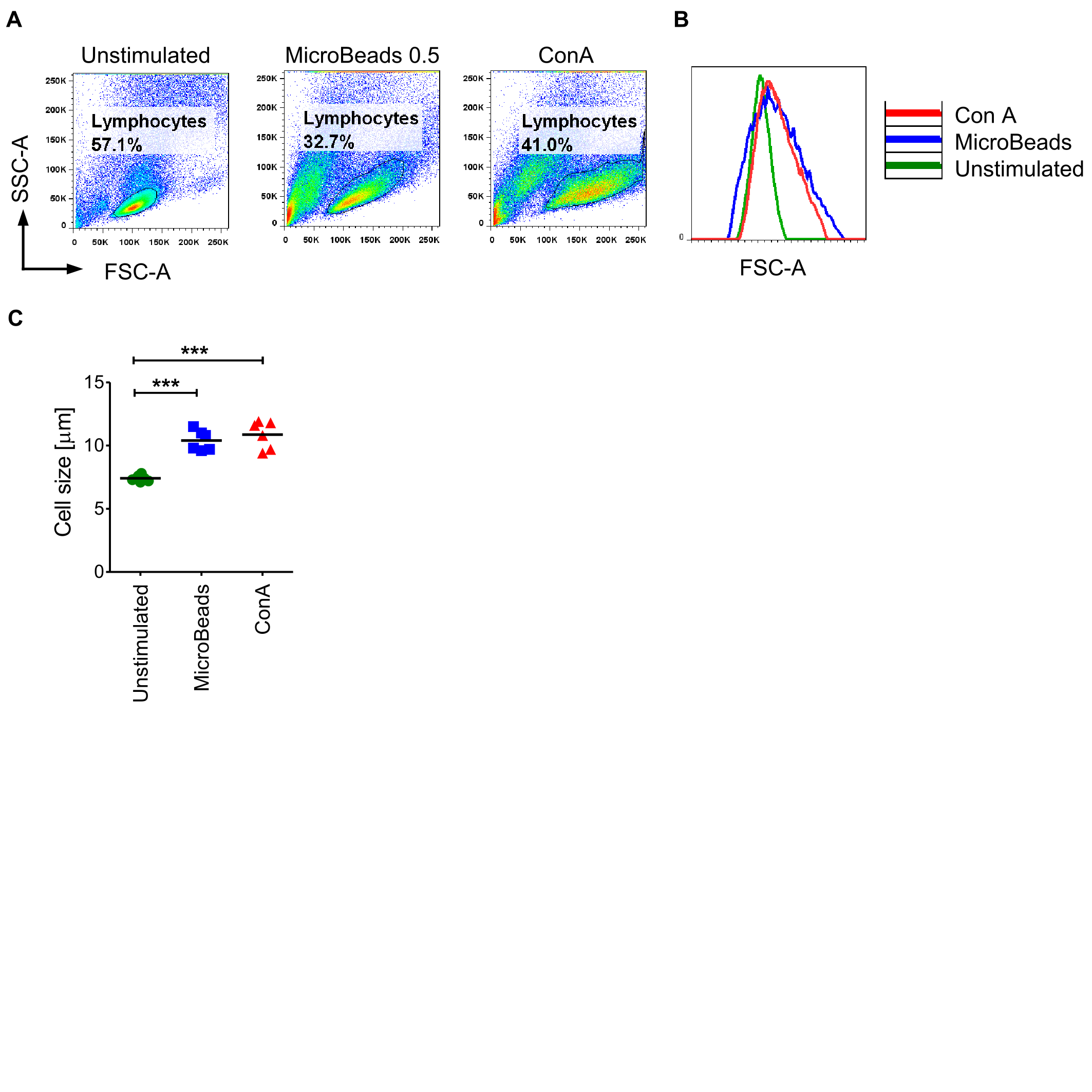

Supplement: Supplementary Figure 1 — Activation with MicroBeads as well as ConA causes an increase in cell size during blast transformation. Representative flow cytometry cytograms (A) and histograms (B) indicating cell size based on FSC scatter. (C) Dot graph showing changes in size of T cells activated with MicroBeads and ConA. Data obtained using Countess II Automated Cell Counter. The average size increased from 7.4 ± 0.22µm in unstimulated cells to 10.4 ± 0.8µm in MicroBeads activated lymphocytes. Data are shown as the mean of six dogs (n=6), and error bars indicate SEM. Statistical analysis was performed by unpaired Student’s t-test (***p < 0.001). [file Image_1.tiff]

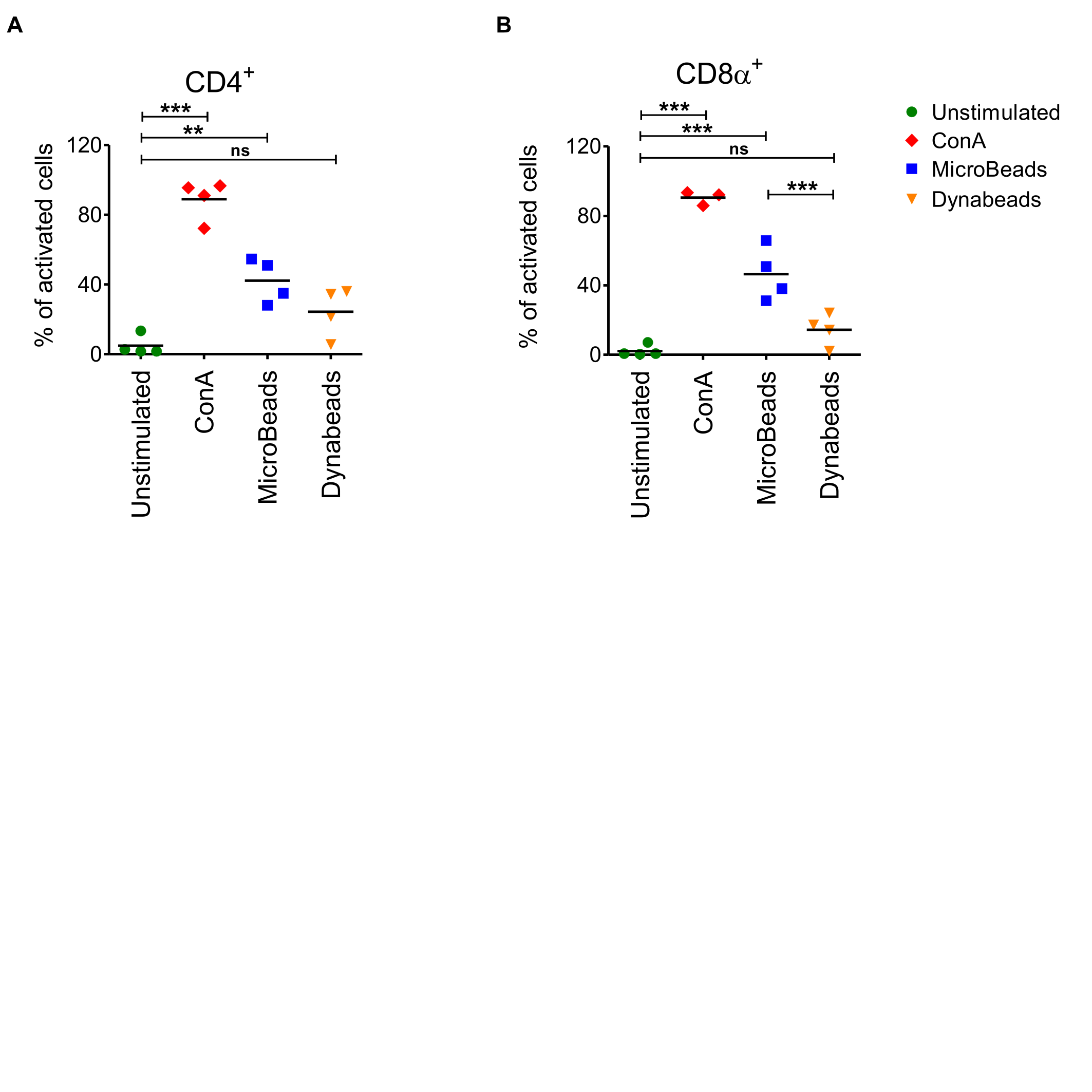

Supplement: Supplementary Figure 2 — Canine T lymphocytes activation efficiency after application of two types of beads (Dynabeads and MicroBeads 1:0.5) coated with anti-canine CD3 and CD28 antibodies. Dot graph showing the percentage of activated CD4+CD25+ (A) and CD8α+CD25+ (B) T cells. Each dot represents one individual dog (n=4). Statistical analysis was performed by One-way analysis of variance (ANOVA) with Tukey’s Multiple Comparison Test (**p < 0.01, ***p < 0.001). [file Image_2.tiff]
